# Reconsidering the ethics of compulsive treatment in light of clinical psychiatry: A selective review of literature

**DOI:** 10.12688/f1000research.109555.3

**Published:** 2022-10-18

**Authors:** Luis Duarte Madeira, Jorge Costa Santos

**Affiliations:** 1 Instituto de Medicina Preventiva, Faculdade de Medicina - Universidade de Lisboa, Lisboa, Lisboa, 1649-035, Portugal; 2 Psiquiatria, CUF Descobertas, Lisboa, 1998-018, Portugal; 3 Instituto Universitário Egas Moniz, Monte de Caparica, 2829-511, Portugal

**Keywords:** involuntary treatment, ethics, persons with disabilities, human rights

## Abstract

The ethics of compulsive treatment (CT) is a medical, social and legal discussion that reemerged after the ratification by 181 countries of the 2007 United Nations Convention on the Rights of Persons with Disabilities (UN-CRPD). The optional protocol of the UN-CRPD was ratified by 86 countries aiming to promote, protect and ensure the full and equal enjoyment of all human rights. It also determined the need to review mental health laws as under this light treatment of persons with disabilities, particularly those with mental disorders, cannot accept the use of CT. This selective review of literature aims to clarify inputs from clinical psychiatry adding evidence to the multi-disciplinary discussion. It provides contradictory evidence on how patients experience CT and its impact on their mental health and treatment programs, also which are main reasons for the use of CT and what efforts in psychiatry have been made to reduce, replace and refine it.

## Introduction

Compulsive treatment ^1^ of people with psychosocial disabilities, particularly when these disabilities result from mental disorders, is a problem of a medical, social, and legal nature. Under the umbrella of Public Health and Health Policies there has been intensive research for legal solutions aiming to settle on the one hand, the need for coercive treatment of people with disabilities who, for various reasons, do not recognize the disorder that affects them (or refuse therapeutic interventions) with the protection of their rights, freedoms and guarantees. Table 1 clarifies some of the historical developments on paternalism and autonomy in the last 50 years.

**Table 1.** Ethical approaches to patients with mental disorders.

Approaches	Description
*Medicalization*	Before the 1980’s: a paternalistic, authoritarian approach in which the physician was free to decide on behalf of the patient “in her/his best interest”
*Legalism* ( Jones 1980)	The existence of legal provisions of external control, particularly of judicial nature, to regulate and safeguard the rights of persons with mental illness
*New legalism* ( Brown 2016)	Aimed to harmonize the procedural safeguards for the provision of adequate health care and treatment of people with psychosocial disabilities in less restrictive conditions

The Council of Europe (CE) and its Bioethics Committee, the European Court of Human Rights (ECHR) promoted several reviews of the mental health legislations. First, the European Convention on Human Rights and Biomedicine (ECHRB), also known as the Oviedo Convention, in 1997 aimed to protect persons that failed to show capacity for consent to treatment (including minors and adults with diminished capacity without representatives) particularly those with mental disorders (article 8) by considering that all medical interventions that could benefit health could be performed under legal provisions in emergency situations (article 8). Second, a new international human rights treaty was drafted in December 2006 and opened for signatures in March 2007 ( Nations 2007) the *United Nations Convention on the rights of Persons with Disabilities*, UN-CRPD. It represents the first comprehensive human rights treaty of the twenty-first century, in force since 2008, and is ratified by 181 countries. Subsequently, several other documents were developed focused on the protection of autonomy, agency and dignity of persons with psychosocial disabilities of which Annual report of the United Nations High Commissioner on Human Rights A/HRC/34/32 is recent example.

The UN-CRPD aims to “promote, protect and ensure the full and equal enjoyment of all human rights and fundamental freedoms by all persons with disabilities” ( Nations 2007, p. 4) and therefore provides an opportunity to discuss and review the ethical foundations of the treatment of persons with mental disorders. Table 2 highlights significant articles from the UN-CRPD.

**Table 2.** Significant article ideas from the United Nations Convention on the rights of Persons with Disabilities.

CRPD articles	Description
*Article 5*	equality and no discrimination
*Article 12 n° 3*	take appropriate measures to support patients with disabilities’ needs to legally exercise their capacity
*Article 12 n° 4*	provide the appropriate and effective safeguards to prevent their abuse, guaranteeing and respecting the rights, will and preference of the person
*Article 14 n° 1b*	ensure that persons with disabilities are not unlawfully or arbitrarily deprived of their liberty, that any deprivation of liberty is in accordance with the law and that the existence of a disability shall in no case justify the deprivation of liberty
*Article 14 n° 2*	warrant that if they are deprived of their liberty, they are entitled to guarantees in accordance with international human rights law and are treated in accordance with objectives and principles contained therein
*Article 15*	Freedom from torture or cruel, inhuman or degrading treatment or punishment
*Article 17*	right to physical and mental integrity

All these provisions are especially important as they imply that disabilities *shall in no case justify the deprivation of liberty* and that competence should be considered at all times – third parties only supporting organization and communication of their will. A literal interpretation of these ideals would determine the immediate interruption of the use of coercive measures in the field of psychiatry, particularly compulsive treatment (CT), for they would consist in a violation of the rights of patients (forcing treatment, discriminating, and marginalizing them). Yet there are several clinical situations that show that providing full autonomy to patients with mental disorders would be devastating – e.g., patients with dementia (unable to manage themselves or their property), with depressive episodes (with suicidal ideation and risk) and psychotic episodes (refusing to feed themselves because they believe they are being poisoned). Indeed, the coercion of persons who can choose (disrespecting autonomy) should be measured against the obligation to make a choice when unable to do so (disrespecting vulnerability). Arguments are raised considering the necessary changes in the restrictions of rights of patients with mental disorders and when it would be ethically and clinically reasonable to objectively limit their autonomy. A powerful/convincing argument against the CRPD effectiveness refers to the fact that its predictions focus on autonomy and are silent on how to effectively determine the duty to protect persons with disabilities and on how to provide adequate health care for those with a severe mental disorder. The general worries about the CRPD are included in Table 3.

**Table 3.** The limitations of the CRPD.

Limitations	Examples
*The misinterpretation of concepts*	e.g. concept of disability can include or not mental disorders whether they are considered extensively or restrictively
*The literal interpretation of measures*	e.g., a simplistic reading of the CRPD determines that “everyone has the right to all the rights and freedom without distinction of any kind" ( Nations 2007, p. 1) jeopardizing the principles of beneficence and of justice when danger is considered to his life or the life of others ( Steinert 2017)
*The risk of acting blindly without considering the complexity*	ethical debate must consider the actual implications in each setting at each stage of its implementation ( Mahomed *et al.* 2018) with scenarios adapted to regional and countrywide circumstances ( Dawson 2015). A protection of the rights of persons ( McSherry and Wilson 2015) leads to a slow and progressive transformation of the healthcare policies and systems

The use of the CRPD should avoid two extreme positions on CT for mental disorders: (1) continuing with the coercive measures acting while considering the “best interest of the patient” and sustaining the proportionality due to “adverse consequences” or the risk of “serious and imminent damage” or (2) determining the immediate abolition of all forms of coercive treatment as its radical reduction is insufficient.

This is a review of literature which provides inputs from psychiatric practice that could clarify how CT is used and felt in the life of patients and health professionals. Particularly, empirical evidence on the uses (and eventual abuses) of CT, of the negative (and possible positive) experience of coercion and of the present ways to reduce and refine CT.

## Methodology

The most relevant journals in the fields of psychiatry (World Psychiatry, The Lancet Psychiatry, Annual Review of Clinical Psychology, JAMA Psychiatry and American Journal of Psychiatry) and medical ethics (BMC Medical Ethics, BMJ Journal of Medical Ethics, Bioethics, American Journal of Bioethics and Journal of Bioethical Inquiry) were searched aiming to achieve full coverage of the topic: “how involuntary commitment is considered in clinical psychiatry?”. The search strategy also included the electronic databases: PubMed, SCOPUS, CINAHL, Web of Science. In PubMed included the use of MESH terms “coercion”, “involuntary commitment” and “Psychiatry” citation tracking and checking of identified eligible articles reference lists were checked for additional articles, and those that are eligible will be included. As inclusion criteria we considered original papers that answered to the use of Involuntary commitment in Clinical Psychiatry. Our search found a total of 893 papers which were examined by two experts (L.M. and J.C.S.) and, after joint discussion, we included 75 papers for review. Search was performed until December 2018. The references identified were merged and managed with Mendeley. Grey literature was not included.

## The use of CT in daily practice

Decisions on coercive measures and on compulsive treatment (CT) appear in the reviewed literature supported by four main reasons: risk, diagnosis, lack of capacity and the effectiveness of the measures. Risk reduction is such a critical factor in the context of compulsive treatment (both in the beginning and interruption) that the measure is perceived as a risk control mechanism ( Hsieh *et al.* 2017). Risk in psychiatry has several dimensions and is subject to qualitative and quantitative assessment – risk of harm to oneself, harm to other persons, of greater social adversity, of suffering more or of compromising a treatment plan ( Light *et al.* 2015).

Risk and diagnosis are fundamental in the decision of CT, as evidence suggests that persons with severe mental disorder can attack and harm others, including health professionals ( Steinert and Traub 2016). Even after CT 25% of patients admitted to an adult psychiatry unit could be at risk of committing an act of violence during their stay at the hospital ( Iozzino *et al.* 2015). Yet the use of “risk of violence” to justify CT is not only the most liable to abuse but also no predictive value of any of the usual individual risk factors for violent behavior – male, diagnosis of schizophrenia, substance abuse and previous history of violence – was found ( Menculini *et al.* 2018). Due to the subjective nature of risk in psychiatry CT must not become a control mechanism for social risk situations and clinicians should bear in mind that it serves to offer treatment to persons with mental disorders ( Rotvold and Wynn 2015a, 2015b, 2016).

Psychotic episodes and behavioral disturbances in patients under previous psychiatric care are most often associated with CT and the symptom profile includes activation, resistance to treatment and “positive” symptoms ( Mosele *et al.* 2018), risk of suicide and low *insight* ( Braitman *et al.* 2014, Masood *et al.* 2017). Evidence for the clinical rational for CT is detailed in Table 4.

**Table 4.** Most frequent reason for compulsive treatment (CT) in clinical practice ( Pignon *et al.* 2014).

**Depressive & mixed Episode**	risk of suicide
**Manic episode**	lack of insight
**Substance abuse**	risky behavior
**Eating disorders**	refuse of support and risk of death
**Personality disorder**	severity of symptoms
**Dementia**	behavioral disturbances

For each country there are precise legal requirements which might not be under use as an analysis of clinical documentation of patients under CT showed that more than 40% clinical records were void of the necessary requirements ( Godet and Niveau 2018). These clinical findings are worrying from an ethical and legal point of view where risk/hazard criteria could lead to dismissal of the need for a diagnosis or for treatment ( Carabellese *et al.* 2017) and also increase prejudice and negative social representations of CT ( Curley *et al.* 2016). The excess of coercive measures in non-Caucasian patients ( Henderson *et al.* 2015) or gender disparities ( Curley *et al.* 2016) furthers the need to explore the motivations for CT. Particularly the risk of coercion ( Rotvold and Wynn 2015b) from other health professionals, from family or the police ( Sjostrand *et al.* 2015) and organizational ( Sjostrand *et al.* 2015) and financial issues ( Green-Hennessy and Hennessy 2015). We must bear in mind that culture and other social factors are also determinants in the application of CT and countries legislations frequently express the cultural milieu and possible constitutional rights (which differ significantly from western to eastern countries as well as from southern to northern Europe).

The role of decision making for CT is challenging, as psychiatrists must distinguish between signs and symptoms of psychiatric disorders (which would determine the use of CT) and those representing behavioral disturbances resulting from a medical condition (no ground for CT). Disregarding such differences might increase the stigma of mental disorders and awareness of them improves the moral weight and reduces random interpretations of clinical practice ( Fistein *et al.* 2016). If CT is to be considered for both then perhaps segregating their legal features is valuable (1) *Medical Incapacity Hold* (MIH) to those who are considered unable to decide and have a psychiatric illness, and (2) *Involuntary Psychiatry Hold (IPH)* for patients with psychiatric disease without *insight* and in need of treatment ( Heldt *et al.* 2018). This separation might allow health professionals to better understand the nature of CT in Psychiatry and distinguish it from the lack of competence in many medical illnesses that confusional states bring about. Drug and addiction disorders have other ethical challenges and while they are clear disturbances of behavior they do not fit into key features of mental disorders and also don’t apply to “medical” incapacities ( Williams 2015).

CT for public health issues, such as tuberculosis, is of particular complexity and represents perhaps the clearest violation of the rights to liberty and privacy in persons whose competence might be unspoiled. Arguments which forward it stand upon both the inviolability of human life (e.g., supporting the coercive use of helmets or forced recycling) and the *principle of reciprocity* – the social obligation as an individual if a group is exposed to detrimental effects on their health resulting from spreading of diseases. This has also supported complex moral decision making under Sars-COV-2 (COVID 19) – e.g. mandatory vaccination. Yet CT for public health reasons has had several censures ( McLaren *et al.* 2016) and other strategies have been proposed ( Karumbi and Garner 2015). First, promotion of health education, increased access to services and settling socioeconomic and organizational determinants are effective for these situations ( Mburu *et al.* 2016). Second, there is contradicting evidence of its effectiveness ( Nagata *et al.* 2014) and it is rarely used even when there is legislation toward it ( Villalbi *et al.* 2016).

The impact of CT in the treatment process should also be measured. Eating disorders (ED) are a good example of the complexity of CT considering clinical severity, capacity to decide, overall risk and effectiveness of the measure. First ED patients don’t seem to have lost the capacity to decide and decision stands upon risk of death ( Westmoreland *et al.* 2017), severity, comorbidities, previous admissions, the incidence of self- injurious behavior ( Clausen and Jones 2014) and yet it might damage therapeutic alliance ( Douzenis and Michopoulos 2015) and lead to early drop-out from other programs ( Schreyer *et al.* 2016). It is possible that forms of power and control (and the need to regulate them) are not only external to the subject but can rise from within. In such case, the patient would need external help in managing, building, and applying decision making or else suffer internal coercion. Internal forms of coercion would then be mediated in the clinical relation in which directivity and surrogate decision making might be helpful. Table 5 shows other contradicting evidence on the effects of CT.

**Table 5.** Contradicting evidence of compulsive treatment (CT).

CT leading to physical and emotional losses in the patient and health team ( Gerace *et al.* 2015)
CT positively impacts on hostility and suicide attempts in psychosis ( Nitschke *et al.* 2018)
CT improves prognosis if there is a high risk of recurrence ( Lera-Calatayud *et al.* 2014)
CT has scarce long-term benefits ( Giacco *et al.* 2018)
CT increases the risk of social adversities and suicide ( Giacco and Priebe 2016)

Experiences of coercion and CT are not one and the same – the first occur in 15% of patients under CT but 20% of patients under voluntary treatment also report coercion ( Edlinger *et al.* 2018). Emotional and cognitive features of the coercive events rather than the number of events appear responsible for its negative impact ( Rusch *et al.* 2014). Such reactions are reduced when patients are allowed to exercise their autonomy, when they experience pleasure in activities/interactions and in the context of a good therapeutic alliance and increased if they endure trauma and humiliation ( Danzer and Wilkus-Stone 2015). Forms of physical coercion appear linked with greater dissatisfaction ( Smith *et al.* 2014, Mielau *et al.* 2016) and therefore medication should be preferred to physical restraint ( Guzman-Parra *et al.* 2018). Yet the evidence isn’t definitive as one study points to involuntary drug administration as the most censored measure ( McLaughlin *et al.* 2016). Moreover, the coercive experience of being under CT might even be linked with dynamic process of recovery – several patients consider that CT was a necessary measure in the end of the treatment ( Gowda *et al.* 2017). Table 6 presents evidence of the negative impact of CT, Table 7 shows evidence on how to reduce these effects ( Opsal *et al.* 2016).

**Table 6.** Evidence on the negative impact of compulsive treatment.

Inducing internalized stigma and lowering adherence ( Kamisli *et al.* 2016)
Prompting experiences of humiliation, oppression and imprisonment ( Nyttingnes *et al.* 2016)
Increasing the length of hospitalization irrespective of severity ( McLaughlin *et al.* 2016)
Damaging the therapeutic alliance increasing the risk of future coercion ( Danzer and Wilkus-Stone 2015)

**Table 7.** Ways to reduce the negative impact of compulsive treatment.

Increasing freedom in all choices still available (e.g., choosing food) ( Danzer and Wilkus-Stone 2015)
Promoting the feeling of physical security (designing and organizing wards for the purpose) ( Lamanna *et al.* 2016)
Training of health professionals towards using non-invasive measures ( Krieger *et al.* 2018)
Considering advanced directives in early stages of the disease ( Mitrossili 2014)
Promoting employment or early return to work ( Pridham *et al.* 2018)
Stimulating empowerment of patients and developing autonomy ( Danzer and Wilkus-Stone 2015)

A range of measures have aimed to reduce the use of coercion and CT ( Kelly *et al.* 2018) either by quantitatively reducing it, by replacing harsher measures or by modifying the experience of coercion. Table 8 indicates these three sorts of changes.

**Table 8.** Measures to reduce, replace and refine compulsive treatment (CT).

**Directly reducing CT**
Crisis centered psychoeducation and crisis cards and 24-month monitoring after inpatient discharge in situations with social difficulties or history of previous hospitalizations ( Lay *et al.* 2018)
Intervention in high-risk states including screening and early intervention ( Santillanes *et al.* 2017)
Multidisciplinary care networks, and community support (including home treatment) all shown to reduce CT and favor early discharge from CT ( Otsuka 2014, Hoffmann *et al.* 2017)
**Replacing CT**
*Community treatment orders* (CTO) and other outpatient settings ( Hansen *et al.* 2018)
*Care in other forms* and *Compulsory community care* ( Reitan 2016)
Short hospital unattended leaves ( Kisely *et al.* 2017)
*Assisted Ambulatory Treatment* (AAT) *and Assertive Community Treatment* (ACT) ( Wullschleger *et al.* 2018)
Crisis teams with training in psychiatry that could carry out/accompany emergency care ( Aagaard *et al.* 2017)
**Refining the use of CT**
Developing non-clinical skills to allow a satisfactory therapeutic alliance ( Jaeger *et al.* 2014)
The use of specific treatment settings, methods and strategies ( Ose *et al.* 2018)
Improving family interventions and social support ( Jong *et al.* 2014, 2016)
Increasing compassion across interventions ( Lamanna *et al.* 2018)
Advance directives (AD) might help ethical challenges in crisis ( Jong *et al.* 2016, Henderson *et al.* 2017)

All these interventions have not received full empirical support due to several contradictory studies. There is paradoxical evidence, such as negative effect of social support ( Hengartner *et al.* 2016) or positive impact of assertive treatments ( Schottle *et al.* 2014, 2018). Moreover, Community Treatment Orders have shown to reduce mortality (9%) and the risk of self-inflicted damage (32%) and provide a modest improvement in the quality of life ( Segal *et al.* 2017) while also requiring large and continued engagement to avoid worse consequences ( Kisely and Campbell 2014, Riley *et al.* 2014). Another paradox is the fact that CT in inpatient settings appears to be better regulated as other team members and patients can supervise what is happening to the patient in comparison with outpatients settings such as Community Treatment Orders ( Riley *et al.* 2014).

## Conclusion

The CRPD addressed the issue of autonomy and decision making by patients with mental disorders determining that alternative solutions to CT must be considered when patients can’t perform responsible decisions. Yet health is a fundamental right and CT offers a protection from hazard which dissolution does not seem to solve – affirming autonomy by conventional ethical models or simplistic clinical approaches ( Kendall 2014) might damage other rights and dignity of persons with mental disorders ( Kelly 2014). Ultimately there is empirical evidence that clinical psychiatry has aimed to clarify the uses and possible abuses of CT, to determine experiences and consequences of its use and developed strategies to reduce, refine and replace it. While there might be the need for interruption of CT in clinical psychiatry, the measures taken cannot risk the misinterpretation of concepts and ignoring the complexity of clinical practice and the systemic changes that should be required. Even if those responsible for CRPD might have already acknowledged these efforts and evidence, while translating these principles into practice stakeholders at nationwide discussions and decisions at a macro level (and possible mental health policies reformations) would benefit from recognizing these inputs from clinical psychiatry where CT takes place.

## Data availability

No data are associated with this article.

## Footnotes

^1^
Scientific literature uses the words “involuntary” (more frequently) or “compulsory” to qualify for hospitalization and/or psychiatric treatment (s) without the informed consent of the mentally ill patient. S/He is considered unable to express autonomously either because s/he refuses to adopt such measures restricting her/his freedom of action, or because s/he does not recognize the disease by which s/he is affected and, consequently, the need for treatment. In this review we chose to favor the term “compulsive”, not only because this is what appears in the relevant legal texts, but also because it is the one that facilitates the communication between the various actors in the process (jurists, doctors, law enforcement and family members). (translated from Lei n.° 36/98, de 24 de Julho (Lei de Saúde Mental), accessible in https://www.pgdlisboa.pt/leis/lei_mostra_articulado.php?nid=276&tabela=leis&so_miolo= **ou** em CEJ: *Internamento Compulsivo.* Lisboa, Coleção Formação Inicial, 2016, acessível em http://www.cej.mj.pt/cej/recursos/ebooks/civil/eb_Internamento_Compulsivo.pdf.
