# Supplementary material for: Reconsidering the ethics of compulsive treatment in light of clinical psychiatry: A selective review of literature
Source: F1000Res. 2022 Oct 18;11:219. Originally published 2022 Feb 23. [Version 3] doi: 10.12688/f1000research.109555.3 (PMC9617066; doi:10.12688/f1000research.109555.3)
Supplement: Peer review history

# Peer review: Reviewer response for version 3

**DOI:** 10.5256/f1000research.138810.r153539

**Published:** 2022-10-28

**Authors:** Sofie Heidenheim Christensen

**Affiliations:** 1 Child and Adolescent Mental Health Center, Copenhagen University Hospital – Mental Health Services CPH, Copenhagen, Denmark

no further comments

Is the review written in accessible language?

Yes

Are all factual statements correct and adequately supported by citations?

Yes

Are the conclusions drawn appropriate in the context of the current research literature?

Yes

Is the topic of the review discussed comprehensively in the context of the current literature?

Yes

Reviewer Expertise:

Treatment in mental health care, including coercive treatment, specifically focused on users' and next of kin's experiences of treatment

I confirm that I have read this submission and believe that I have an appropriate level of expertise to confirm that it is of an acceptable scientific standard.

# Peer review: Reviewer response for version 2

**DOI:** 10.5256/f1000research.136140.r147228

**Published:** 2022-09-13

**Authors:** Sofie Heidenheim Christensen

**Affiliations:** 1 Child and Adolescent Mental Health Center, Copenhagen University Hospital – Mental Health Services CPH, Copenhagen, Denmark

**Summary:**

 The selective literature review adresses the problem of coercive treatment (CT) in mental health, mainly the juxtaposition of coercion as a violation of rights with the clinical obligation to treat patients with mental disorders.

 Firstly, the practice and impact of CT is presented with focus on risk asessment, impact on treatment. Secondly, the experience of coercion and how this may be ameliorated is shortly presented. And lastly, the article touches upon ways to reduce or ameliorate CT.

**Critique and suggestions:**

 The focus on clinical inputs with regards to the ethical dilemma of CT is an important perspective. The very literal interpretation of the articles from the United Nations Convention on the rights of Persons with Disabilities seems not sufficiently nuanced - all articles cited in Table 2 provides caveats. If possible, nuance this point in the conclusion, as I am not, from the material provided, sufficiently convinced that stakeholders do not in fact "acknowledge these efforts and evidence"

 "This is a review of literature which provides inputs from psychiatric practice..." – The article does not specify what literature has been reviewed and how the is sample created, and would benefit greatly from this tranparency in terms of validity.

 Clear tables, that neatly summarise the points of the article. Very nice.

 "Even after CT 25% of patients admitted to an adult psychiatry unit could be at risk of committing an act of violence" – Please clarify, after CT has ended, during CT?

 "Yet while individual risk factors appeared for violent behaviors and CT decisions ( Menculini *et al.* 2018) – e.g. male, diagnosis of schizophrenia, substance abuse and previous history of violence – their predictive value for violent behavior was not found. Of all the reasons the risk of occurrence or recurrence of violence seems the most liable to abuse (due to the subjective nature of risk in psychiatry) and perhaps the target of the CRPD worries and predicaments – that a measure aimed at the treatment of persons with mental disorders becomes a control mechanism for social risk situations. Indeed, some argue that it overlaid the true reason of CT need for treatment" – This argument is unclear.

 "Yet for each country there are legal requirements which might not be under use – more than 40% patients failed to provide them in their records" – Did the patients fail to provide them or did the hospital/administration? Please clarify.

 "This would allow health professionals to" – The rest of the sentence seems to be missing.

 "CT for public health issues, such as tuberculosis, is of particular complexity and represents perhaps the clearest violation of the rights to liberty and privacy in persons whose competence might be unspoiled." – It seems strange to bring up CT for public health issues in an article focused on mental health, and, if this is a deliberate choice, without a mention of COVID and the moral dilemmas posed by the pandemic.

 "Postmodern ethics suggest that forms of power and control (and the need to regulate them) are not only external to the subject but can rise from within." – Citation missing?

 "When they experience satisfaction" – With what?

 "Several patients admit" – Admit is a loaded concept (linked to admission of guilt), consider a more neutral “report“ or “consider”.

 "Another paradox is the fact that CT in inpatient settings appears to be better regulated as other team members and patients can supervise what is happening to the patient" – To clarify, add “in comparison with Community Treatment Order”.

 "interruption of forms of CT" – of certain forms? Be specific as to which forms.

**Conclusion:**

 An interesting perspective, with relevant insights from clinical practice, that would benefit from a section on methodology.

Is the review written in accessible language?

Yes

Are all factual statements correct and adequately supported by citations?

Yes

Are the conclusions drawn appropriate in the context of the current research literature?

Yes

Is the topic of the review discussed comprehensively in the context of the current literature?

Yes

Reviewer Expertise:

Treatment in mental health care, including coercive treatment, specifically focused on users' and next of kin's experiences of treatment

I confirm that I have read this submission and believe that I have an appropriate level of expertise to confirm that it is of an acceptable scientific standard, however I have significant reservations, as outlined above.

# Response

**Published:** 2022-09-18

**Authors:** Luis Madeira

Answers to the reviewer

**The selective literature review adresses the problem of coercive treatment (CT) in mental health, mainly the juxtaposition of coercion as a violation of rights with the clinical obligation to treat patients with mental disorders.**

**Firstly, the practice and impact of CT is presented with focus on risk asessment, impact on treatment. Secondly, the experience of coercion and how this may be ameliorated is shortly presented. And lastly, the article touches upon ways to reduce or ameliorate CT.**

**Critique and suggestions:**

***The focus on clinical inputs with regards to the ethical dilemma of CT is an important perspective. The very literal interpretation of the articles from the United Nations Convention on the rights of Persons with Disabilities seems not sufficiently nuanced - all articles cited in Table 2 provides caveats. If possible, nuance this point in the conclusion, as I am not, from the material provided, sufficiently convinced that stakeholders do not in fact "acknowledge these efforts and evidence"***

 ANSWER: We thank the reviewer for pinpointing this possibility and although the CRPD commission to draft the final document did not include any doctors (who could attest for the difficulties of dealing with a simple legislation that does not encompass the nuances of clinical practice) we’ve now changed the last sentence of the paper which now reads “ *Even if those responsible for CRPD might have already acknowledged these efforts and evidence, while translating these principles into practice* *stakeholders at nationwide discussions and decisions at a macro level (and possible mental health policies reformations) would benefit from* *recognizing these inputs from clinical psychiatry where CT takes* *actually takes place.* *”*

**"This is a review of literature which provides inputs from psychiatric practice..." – The article does not specify what literature has been reviewed and how the is sample created, and would benefit greatly from this tranparency in terms of validity.**

 ANSWER: We agree with the reviewer that this is a major flaw of our paper. This paper is a review of the papers used to produce the national assembly document for Compulsive Treatment in Portugal as we were the most active participants to develop a new mental health law. So, for the first task we both used research methodology and while reviewing the document we did not considered the methodology – we have now salvaged the methodology so that it strengthen the quality of our manuscript. The methodology now reads: The most relevant journals in the fields of psychiatry (World Psychiatry, The Lancet Psychiatry, Annual Review of Clinical Psychology, JAMA Psychiatry and American Journal of Psychiatry) and medical ethics (BMC Medical Ethics, BMJ Journal of Medical Ethics, Bioethics, American Journal of Bioethics and Journal of Bioethical Inquiry) were searched aiming to achieve full coverage of the topic: “how involuntary commitment is considered in clinical psychiatry?”. The search strategy also included the electronic databases: PubMed, SCOPUS, CINAHL, Web of Science. In PubMed included the use of MESH terms “coercion”, “involuntary commitment” and “Psychiatry” citation tracking and checking of identified eligible articles reference lists were checked for additional articles, and those that are eligible will be included. As inclusion criteria we considered original papers that answered to the use of Involuntary commitment in Clinical Psychiatry. Our search found a total of 893 papers which were examined by two experts (L.M. and J.C.S.) and, after joint discussion, we included 75 papers for review. Search was performed until December 2018. The references identified were merged and managed with Mendeley. Grey literature was not included.

**Clear tables, that neatly summarise the points of the article. Very nice.**

 ANSWER: We thank the reviewer for the positive remark about the paper.

**"Even after CT 25% of patients admitted to an adult psychiatry unit could be at risk of committing an act of violence" – Please clarify, after CT has ended, during CT?**

 ANSWER: We thank the reviewer to identify the lack of clarification of this sentence – this is an important clarification. The sentence now reads “after CT 25% of patients admitted to an adult psychiatry unit could be at risk of committing an act of violence during their stay at the hospital”

**"Yet while individual risk factors appeared for violent behaviors and CT decisions ( Menculini *et al.* 2018) – e.g. male, diagnosis of schizophrenia, substance abuse and previous history of violence – their predictive value for violent behavior was not found. Of all the reasons the risk of occurrence or recurrence of violence seems the most liable to abuse (due to the subjective nature of risk in psychiatry) and perhaps the target of the CRPD worries and predicaments – that a measure aimed at the treatment of persons with mental disorders becomes a control mechanism for social risk situations. Indeed, some argue that it overlaid the true reason of CT need for treatment" – This argument is unclear.**

 ANSWER: We thank the reviewer for identifying how difficult it is to retrieve the main argument of this paragraph. It was extensively edited, and we hope it is now clearer. It now reads: “Yet the use of “risk of violence” to justify CT is not only the most liable to abuse but also no predictive value of any of the usual individual risk factors for violent behavior - male, diagnosis of schizophrenia, substance abuse and previous history of violence - was found ( Menculini *et al.* 2018). Due to the subjective nature of risk in psychiatry CT must not become a control mechanism for social risk situations and clinicians should bear in mind that it serves to offer treatment to persons with mental disorders”

**"Yet for each country there are legal requirements which might not be under use – more than 40% patients failed to provide them in their records" – Did the patients fail to provide them or did the hospital/administration? Please clarify.**

 ANSWER: We thank the reviewer for identifying a necessary change to better present the data involved. It now reads “For each country there are precise legal requirements which might not be under use as an analysis of clinical documentation of patients under CT showed that more than 40% clinical records were void of the necessary requirements”

**"This would allow health professionals to" – The rest of the sentence seems to be missing.**

 ANSWER: We’re very sorry that this has happened – this sentence was edited during the first reviewer comments and became incomplete – “This separation might allow health professionals to betterunderstand the nature of CT in Psychiatry and distinguish it from the lack of competence in many medical illnesses that confusional states bring about.”

**"CT for public health issues, such as tuberculosis, is of particular complexity and represents perhaps the clearest violation of the rights to liberty and privacy in persons whose competence might be unspoiled." – It seems strange to bring up CT for public health issues in an article focused on mental health, and, if this is a deliberate choice, without a mention of COVID and the moral dilemmas posed by the pandemic.**

 ANSWER: We thank the reviewer for finding this incongruence. Yet this follows from the reviewed literature and seemed to be along with the possibility of (mal)using CT in psychiatry as a social risk measure. We didn’t include the situation with Sars-COV-2 (COVID 19 pandemic) as the reviewed papers were published until 2019 and made no reference to it. Yet we agree that the paper, being published in 2022, should include them so we now edited the paragraph to read: “Arguments which forward it stand upon both the inviolability of human life (e.g., supporting the coercive use of helmets or forced recycling) and the principle of reciprocity – the social obligation as an individual if a group is exposed to detrimental effects on their health resulting from spreading of diseases. This has also supported complex moral decision making under Sars-COV-2 (COVID 19) – e.g. mandatory vaccination.”

**"Postmodern ethics suggest that forms of power and control (and the need to regulate them) are not only external to the subject but can rise from within." – Citation missing?**

 ANSWER: We thank the reviewer for finding this typo. This was also an edited sentence during the first review of the manuscript that was copied with the citation marks but is a sentence of the authors. It is now corrected “It is possible that forms of power and control (and the need to regulate them) are not only external to the subject but can rise from within.”

**"When they experience satisfaction" – With what?**

 ANSWER: We thank the reviewer for identifying the need of further clarification. We went through the original paper and found that the correct word is pleasure – when they are pleased with activities / interactions during the CT. It now reads “Such reactions are reduced when patients are allowed to exercise their autonomy, when they experience pleasure in activities/interactions”

**"Several patients admit" – Admit is a loaded concept (linked to admission of guilt), consider a more neutral “report“ or “consider”.**

 ANSWER: We completely agree with the reviewer that admit is not the accurate word for this sentence – we’ve replaced with consider as suggested.

**"Another paradox is the fact that CT in inpatient settings appears to be better regulated as other team members and patients can supervise what is happening to the patient" – To clarify, add “in comparison with Community Treatment Order”.**

 ANSWER: We agree with the reviewer that the sentence is unclear. It now reads “ *Another paradox is the fact that CT in inpatient settings appears to be better regulated as other team members and patients can supervise what is happening to the patient in comparison with* *outpatients’ settings* *such as Community Treatment Order* *s*”

**"interruption of forms of CT" – of certain forms? Be specific as to which forms.**

 ANSWER: we agree with the reviewer that the word “forms” should not be part of the wording of this sentence or else it would require us to be specific which would extend the conclusion and not add to the overall idea. The sentence now reads: “While there might be the need for interruption of CT in clinical psychiatry, measures taken cannot risk the misinterpretation of concepts and ignoring the complexity of clinical practice and the systemic changes that should be required.”

# Peer review: Reviewer response for version 2

**DOI:** 10.5256/f1000research.136140.r144636

**Published:** 2022-07-27

**Authors:** Mohammadreza Shalbafan

**Affiliations:** 1 Iran University of Medical Sciences, Tehran, Iran

Thank you. No additional comment.

Is the review written in accessible language?

Partly

Are all factual statements correct and adequately supported by citations?

Yes

Are the conclusions drawn appropriate in the context of the current research literature?

Yes

Is the topic of the review discussed comprehensively in the context of the current literature?

Partly

Reviewer Expertise:

Psychiatry, Mental Health, Stigma, COVID-19, OCD, Psychopharmacology, Depression

I confirm that I have read this submission and believe that I have an appropriate level of expertise to confirm that it is of an acceptable scientific standard.

# Peer review: Reviewer response for version 1

**DOI:** 10.5256/f1000research.121068.r142241

**Published:** 2022-07-11

**Authors:** Mohammadreza Shalbafan

**Affiliations:** 1 Iran University of Medical Sciences, Tehran, Iran

The manuscript discusses an important topic and it`s well-written, by and large. I have some additional comments in order to improve the manuscript:

1. Type of the paper should be added to the title.
2. Main findings of the paper should be emphasized in the abstract.
3. 'CRPD' should be replaced with an appropriate key-word from MeSH.
4. '(article 6)' and some others are not clear enough and should be mentioned more clearly.
5. Description of the columns should be added to the tables.
6. The manuscript needs proof-reading, particularly for capitals.
7. What`s 'CTO'?
8. Cultural and trans-cultural aspects of the topic should be discussed briefly.

Is the review written in accessible language?

Partly

Are all factual statements correct and adequately supported by citations?

Yes

Are the conclusions drawn appropriate in the context of the current research literature?

Yes

Is the topic of the review discussed comprehensively in the context of the current literature?

Partly

Reviewer Expertise:

Psychiatry, Mental Health, Stigma, COVID-19, OCD, Psychopharmacology, Depression

I confirm that I have read this submission and believe that I have an appropriate level of expertise to confirm that it is of an acceptable scientific standard, however I have significant reservations, as outlined above.

# Response

**Published:** 2022-07-12

**Authors:** Luis Madeira

Answer to the reviewer,

 We thank the reviewer for going through our paper and provide a critical appraisal of its content which we believe greatly improve its quality. We answer below individually to the changes requested.

 The manuscript discusses an important topic and it`s well-written, by and large. I have some additional comments to improve the manuscript: 

1. Type of the paper should be added to the title.

Answer: We thank the reviewer for considering this add on to the paper. It now reads *“* *Reconsidering the ethics of compulsive treatment in light of clinical psychiatry: **A selective review of literature”***. We also agree that it clarifies the purpose of the paper.

  

1. Main findings of the paper should be emphasized in the abstract.

Answer: We agree with the suggestion of the reviewer. We’ve now edited the abstract though due to limitations in the number of words we’ve greatly abbreviated the main findings of the paper. It now reads “ *The ethics of compulsive treatment (CT) is a medical, social and legal discussion that reemerged after the ratification by 181 countries of the 2007 United Nations Convention on the Rights of Persons with Disabilities (UN-CRPD). The optional protocol of the UN-CRPD was ratified by 86 countries aiming to promote, protect and ensure the full and equal enjoyment of all human rights. It also determined the need to review mental health laws as under this light treatment of persons with disabilities, particularly those with mental disorders, cannot accept the use of CT. This selective review of literature aims to clarify inputs from clinical psychiatry adding evidence to the multi-disciplinary discussion. It provides contradictory evidence on how patients experience CT and its impact on their mental health and treatment programs, also which are main reasons for the use of CT and what efforts in psychiatry have been made to reduce, replace and refine it.”*

  

1. 'CRPD' should be replaced with an appropriate key-word from MeSH.

Answer: We agree with the reviewer that the word should be replaced, and we suggest that it Persons with Mental **Disabilities** which is the closest mesh term.

  

1. '(article 6)' and some others are not clear enough and should be mentioned more clearly.

Answer: We’ve now added extra density to the article 6 which reads “aimed to protect persons that failed to show capacity for consent to *treatment (including minors and adults with diminished capacity without representatives)* (article 6)”

  

1. Description of the columns should be added to the tables.

Answer: We thank the reviewer for pointing this idea as we have now edited the tables and added the description of columns to all tables with 2 or more columns. We believe that single column tables are described by the descriptor above them.

  

1. The manuscript needs proof-reading, particularly for capitals.

Answer: Our manuscript was proof-readed by a paid English-speaking professional translator. Yet we’ve now asked two native English speakers to go through the manuscript again.

  

1. What`s 'CTO'?

Answer: We replaced CTO for Community Treatment Orders – a specific form of compulsive ambulatory treatment. The explanation was in the table but fits better in the text itself as the reader might be unable to reach understand CTO abbreviation.  

  

1. Cultural and trans-cultural aspects of the topic should be discussed briefly.

Answer: This is a very interesting and relevant topic in the field of compulsive treatment. Considering the limitations in the number of words we have included the following paragraph “We must bear in mind that culture and other social factors are also determinants in the application of CT and countries legislations frequently express the cultural milieu and possible constitutional rights (which differ significantly from western to eastern countries as well as from southern to northern Europe).”
